# MED12 exerts an emerging role in actin-mediated cytokinesis via LIMK2/cofilin pathway in NSCLC

**DOI:** 10.1186/s12943-019-1020-4

**Published:** 2019-05-09

**Authors:** Meng Xu, Fang Wang, Guibo Li, Xiaokun Wang, Xiaona Fang, Haoxuan Jin, Zhen Chen, Jianye Zhang, Liwu Fu

**Affiliations:** 1 Sun Yat-sen University Cancer Center, State Key Laboratory of Oncology in South China, Collaborative Innovation Center for Cancer Medicine, No.651 Dongfeng East Road, Guangzhou, 510060 Guangdong China; 2 Radiotherapy Department of Thorax & Abdomen Tumor, Cancer Center, The First People’s Hospital of Foshan Affiliated to Sun Yat-sen University, Foshan, 528000 China; 3 Beijing Genomics Institute (BGI)-Shenzhen, Shenzhen, 518083 China; 4 School of Pharmaceutical Sciences, Guangzhou Medical University, Guangzhou, 511436 China

**Keywords:** MED12, Cytokinesis, Actin, LIMK2, NSCLC

## Abstract

**Background:**

Mediator complex subunit 12 (MED12) is an essential hub for transcriptional regulation, in which mutations and overexpression were reported to be associated with several kinds of malignancies. Nevertheless, the role of MED12 in non-small cell lung cancer (NSCLC) remains to be elucidated.

**Methods:**

*MED12* mutation was detected by Next-generation sequencing. The expression of MED12 in 179 human NSCLC tissue samples and 73 corresponding adjacent normal lung tissue samples was measured by immunohistochemistry (IHC). CRISPR-Cas9 was used to knock out MED12 in PC9 and SPC-A1 cells. MED12 rescued stable cell lines were generated by lentivirus infection. We traced cell division process by live cell imaging. The molecular mechanism of aborted cytokinesis resulted by MED12 knockout was investigated by RNA-seq. Effects of MED12 deletion on the proliferation of NSCLC cells were determined by MTT assay and Colony-formation assay in vitro and xenograft tumor model in nude mouse. Cell senescence was measured by SA-β-gal staining.

**Results:**

In our study, no *MED12* exon mutation was detected in NSCLC samples, whereas we found that MED12 was overexpressed in human NSCLC tissues, which positively correlated with the tumor volume and adversely affected patient survival. Furthermore, knockout MED12 in NSCLC cell lines resulted in cytokinesis failure, displayed a multinuclear phenotype, and disposed to senescence, and become non-viable. Lack of MED12 decreased the proliferative potential of NSCLC cells and limited the tumor growth in vivo. Mechanism investigations revealed that MED12 knockout activated LIMK2, caused aberrant actin cytoskeleton remodeling, and disrupted the abscission of intercellular bridge, which led to the cytokinesis failure. Reconstitution of exogenous MED12 restored actin dynamics, normal cytokinesis and cell proliferation capacity in MED12 knockout cells.

**Conclusions:**

These results revealed a novel role of MED12 as an important regulator for maintaining accurate cytokinesis and survival in NSCLC cells, which may offer a therapeutic strategy to control tumor growth for NSCLC patients especially those highly expressed MED12.

**Electronic supplementary material:**

The online version of this article (10.1186/s12943-019-1020-4) contains supplementary material, which is available to authorized users.

## Introduction

*MED12* encodes a component of Mediator, a conserved multi-subunit complex implicated in the transcriptional regulation of many genes by mediating the interaction of RNA Polymerase II (Pol II) with gene-specific transcriptional factors [1]. Somatic mutations in this X linked gene impaired MED12 activities and were associated with several tumors, including uterine leiomyoma, breast fibroadenoma and prostate cancer [2–4]. Interestingly, distribution of *MED12* mutation sites differs in different types of tumor. In uterine leiomyomas and breast fibroadenoma, *MED12* mutations were found in the stromal cells and mainly located in the exon 2 region which led to the activation of the WNT pathway [2, 3]. While in prostatic carcinoma, *MED12* mutation sites were identified in exon 26 in the epithelial cells which seem to influence androgen signaling pathway [4]. Additionally, over-expression of MED12 in prostatic carcinoma as well as breast cancer has been observed [5–7]. Knockdown of MED12 in cancer cells led to an apparent cell proliferation defect by arrested cell cycle at G0/G1 phase [5, 8, 9]. Non-small cell lung cancer (NSCLC) as the leading cause of cancer- related death all over the world, the relevance of MED12 in which including mutations, expression and function has not been explored.

Cell division is necessary for cell multiplication which involves an ordered sequence of events: replication of the genome, chromosome segregation, and cytokinesis [10]. Cytokinesis progression in animal cells, including actomyosin cleavage apparatus assemble and efficient midbody abscission: the actomyosin contractile ring was formed once the plasma membrane started to ingress, then the daughter cells moved apart to disclose the intercellular bridge stretched between them, cytokinesis was completed when the intracellular bridge was cut off [11, 12]. Completion of cytokinesis requires temporally and spatially regulated communication from the microtubule cytoskeleton to the actin cytoskeleton and the cell membrane [13, 14]. Among them, actin guaranteed normal completion of actomyosin ring assembly or other cellular motile events through highly dynamic switch between monomeric (G-actin) and filamentous (F-actin) status [14, 15]. G-actin polymerizes in a head-to-tail manner to form helical F-actin and the equilibrium between G-actin and F-actin was tightly regulated by actin-associated proteins including LIMK2 [16]. LIMK2 as a downstream target of the Rho/ROCK pathway, regulated actin dynamics via phosphorylates and inactivates the actin depolymerizing factor cofilin [16, 17]. Disrupted actin dynamics could induce aborted cytokinesis and decrease cell proliferation [18–20]. Recent study indicated that the Mediator complex subunit 8 (Med8) controls actin assembly via bond to Arp2/Arp3 complex [21]. However, the correlation of MED12 with actin assembly has not been explored.

In this study, we provided the first phenotypic and molecular evidence that MED12 was indispensable for actin-mediated cytokinesis via bound to the promoter of LIMK2.

## Results

### MED12 mutation and expression in human NSCLC tissues

**Fig. 1:**
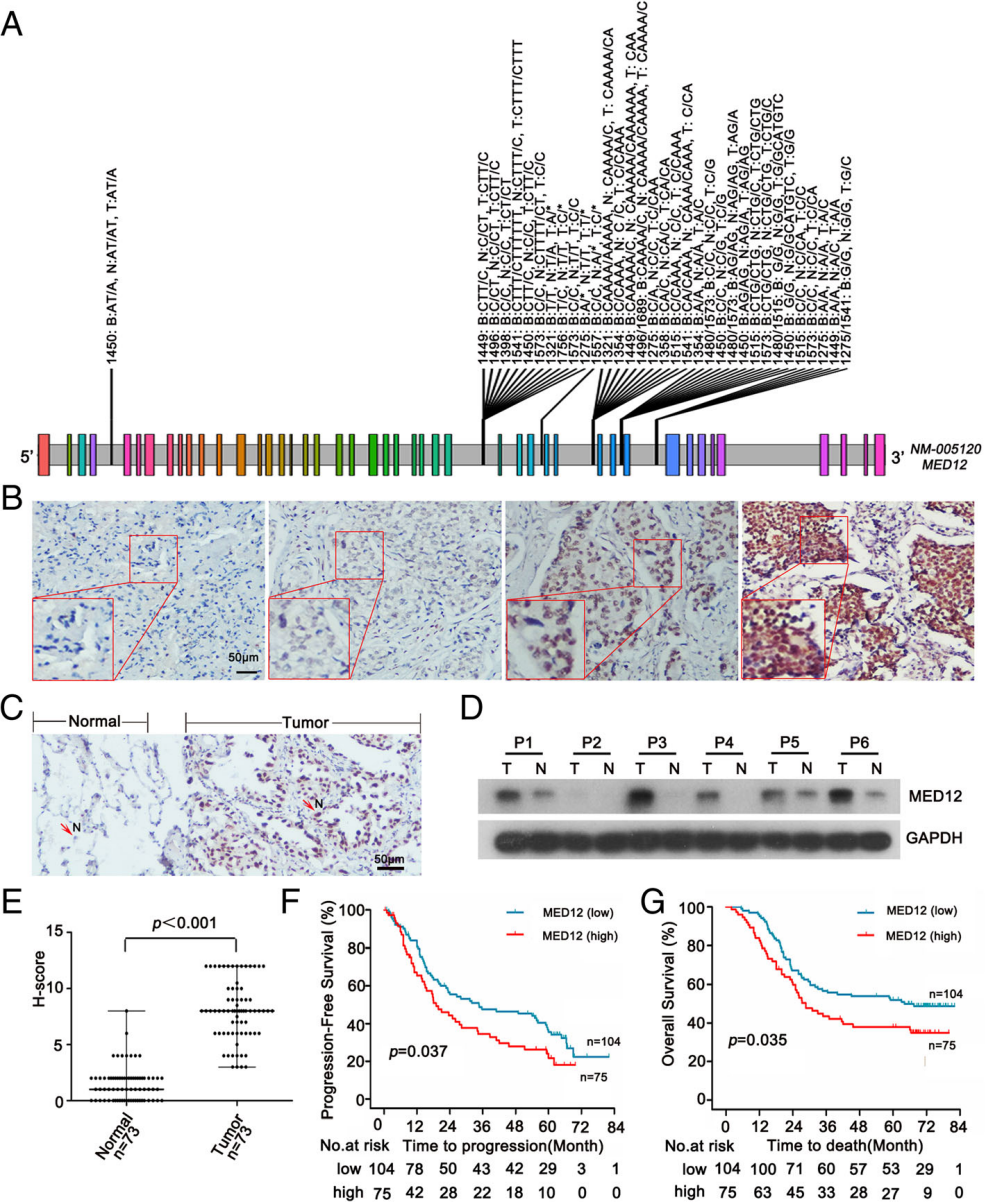
MED12 mutation and expression in human NSCLC tissues. **a** Next-Generation Sequencing consequence of RT-PCR products from 40 samples of NSCLC tissue and blood indicated no mutation at exons of *MED12* gene. Color stripes: exons; gray stripes: introns. **b** Representative images of negative (−), weak (+), moderate (++), strong (+++) MED12 IHC staining in NSCLC tissues. Scale bar: 50 μm. **c** Representative image of MED12 IHC staining in the tumor tissue and adjacent normal lung epithelial tissue. Abbreviation N denoted nuclear location of MED12. Scale bar: 50 μm. **d** Western blotting analysis of MED12 expression in 6 matched NSCLC tumor tissues lysates and normal lung tissues lysates. T: tumor tissue, N: normal lung tissue. **e** Quantification of MED12 expression in 73 samples of tumorous and adjacent normal lung epithelial tissues. **f**, **g**, The Kaplan-Meier plot showed the progression-free survival (PFS) and overall survival (OS) in comparison of patients with high or low MED12 expression

To better understand *MED12* mutational status in human NSCLC, we implemented Next-Generation DNA sequencing on 40 patients with their matching whole-blood samples. We didn’t observe MED12 exon mutation in these patients (Fig. 1a). Next, we examined MED12 protein levels using immunostaining analysis (IHC) in a large panel of NSCLC samples (*n* = 179) and adjacent normal lung tissues (*n* = 73). We found that MED12 was heterogeneously expressed in tumor cells (Fig. 1b). MED12 showed a nuclear expression pattern, and MED12 expression was dramatically higher in NSCLC specimens than in adjacent normal lung tissues (Fig. 1c-e). Moreover, MED12 expression was positively correlated with tumor size (Table 1, *p* = 0.036). Further analysis revealed that patients with high MED12 expression had shorter progression-free survival (PFS) and 5-year overall survival (OS) than those with low MED12 expression (Fig. 1f and g). Taken together, these results demonstrated that MED12 exon mutation was not a common event in NSCLC and elevated MED12 expression was linked to NSCLC malignant growth.

**Table 1:** Correlation between MED12 expression and clinic pathologic parameters in NSCLC

	Overall	MED12 expression level		*p* value
		Low expression	High expression	
No. of patients	179	104	75	
Gender				
Male	118	65	53	0.164
Female	61	39	22	
Age				
≤ 60 ys	104	65	39	0.105
> 60 ys	75	39	36	
Tumor size				
≤ 5 cm	147	91	56	**0.031**
> 5 cm	32	13	19	
Lymph node status				
N0	88	49	39	0.519
N+	91	55	36	
M classification				
M0	169	98	71	0.9
M1	10	6	4	
Clinical stage				
I + II	95	54	41	0.763
III + IV	84	50	34	
Histopathologic type				
Adenocarcinoma	137	78	59	0.568
Non-adenocarcinoma	42	26	16	

Table legend: Higher MED12 expression was strongly correlated with larger tumor size, *p* = 0.031

### MED12 deficiency suppressed NSCLC cell proliferation and xenograft tumor growth

**Fig. 2:**
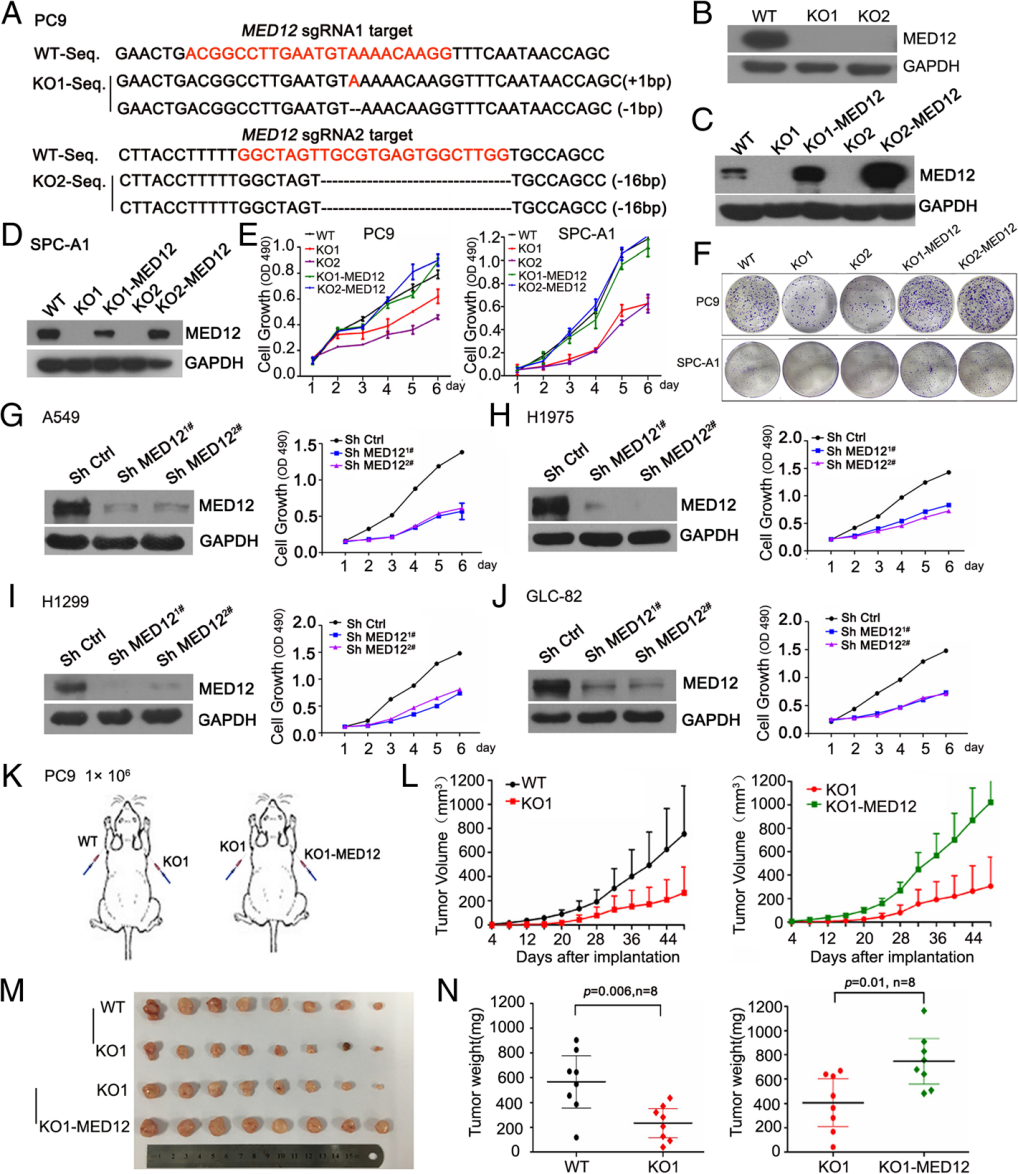
MED12 knockout suppressed cell proliferation and xenograft tumor growth. **a** DNA sequencing validated the indels genotype of two *MED12* alleles in PC9 MED12 knockout clones. **b** Western blotting assay of MED12 in PC9 single clones after using specific *MED12* sgRNAs by CRISPR. **c**-**d** Western blotting assays of MED12 in WT, MED12 KO and MED12 reconstitution single clones of PC9 and SPC-A1 cells. **e** MTT assay showed the proliferation ability of WT, MED12 KO and MED12 reconstitution single clones of PC9 and SPC-A1. **f** Colony formation assays in WT, MED12 KO and MED12 reconstitution single clones of PC9 and SPC-A1 cells. **g**-**j** Efficient knockdown of MED12 in various NSCLC cell lines were revealed by western blotting and cell proliferation examined by MTT assay. **k** Schema of xenograft in vivo assay. **l** Tumor growth curve of WT/KO1 cells (left panel) and KO1/KO1-MED12 cells (right panel) in nude mice. The number of mice in each group was eight. **m** Image of xenografts when nude mice sacrificed. **n** Tumor weight of WT/KO1 group (left panel) and KO1/KO1-MED12 group (right panel)

To determine the biological role of MED12 in NSCLC, we knocked out endogenous MED12 in human lung adenocarcinoma cells PC9 and SPC-A1 using the CRISPR-Cas9 system and reconstitution of exogenous MED12 using Lentiviral system (Fig. 2a-d). We next examined whether MED12 may regulate cancer cell proliferation. The MTT assay showed that MED12 KO clones proliferated significantly slower from day 2 compared with the MED12 WT or MED12 reconstitution single clones (Fig. 2e). This phenomenon was further confirmed by a colony formation assay (Fig. 2f). We also knockdown of MED12 with shRNAs in a few more NSCLC cell lines and found the same results (Fig. 2g-j). To further determine the role of MED12 in cancer development, we performed xenograft tumor growth assay using three stable PC9 clones with or without MED12 expression (Fig. 2k). The data showed that proliferate rate of MED12 KO1 xenograft tumors was significantly lower than WT or KO1-MED12 xenograft tumors (Fig. 2l). Also, significant difference in tumor weight between WT and KO1 or KO1 and KO1-MED12 was observed (Fig. 2m and n). In summary, these in vitro and in vivo data demonstrated that lack of MED12 decreased the proliferative potential of NSCLC cells.

### MED12 knockout caused a multinucleation phenotype in NSCLC cells

**Fig. 3:**
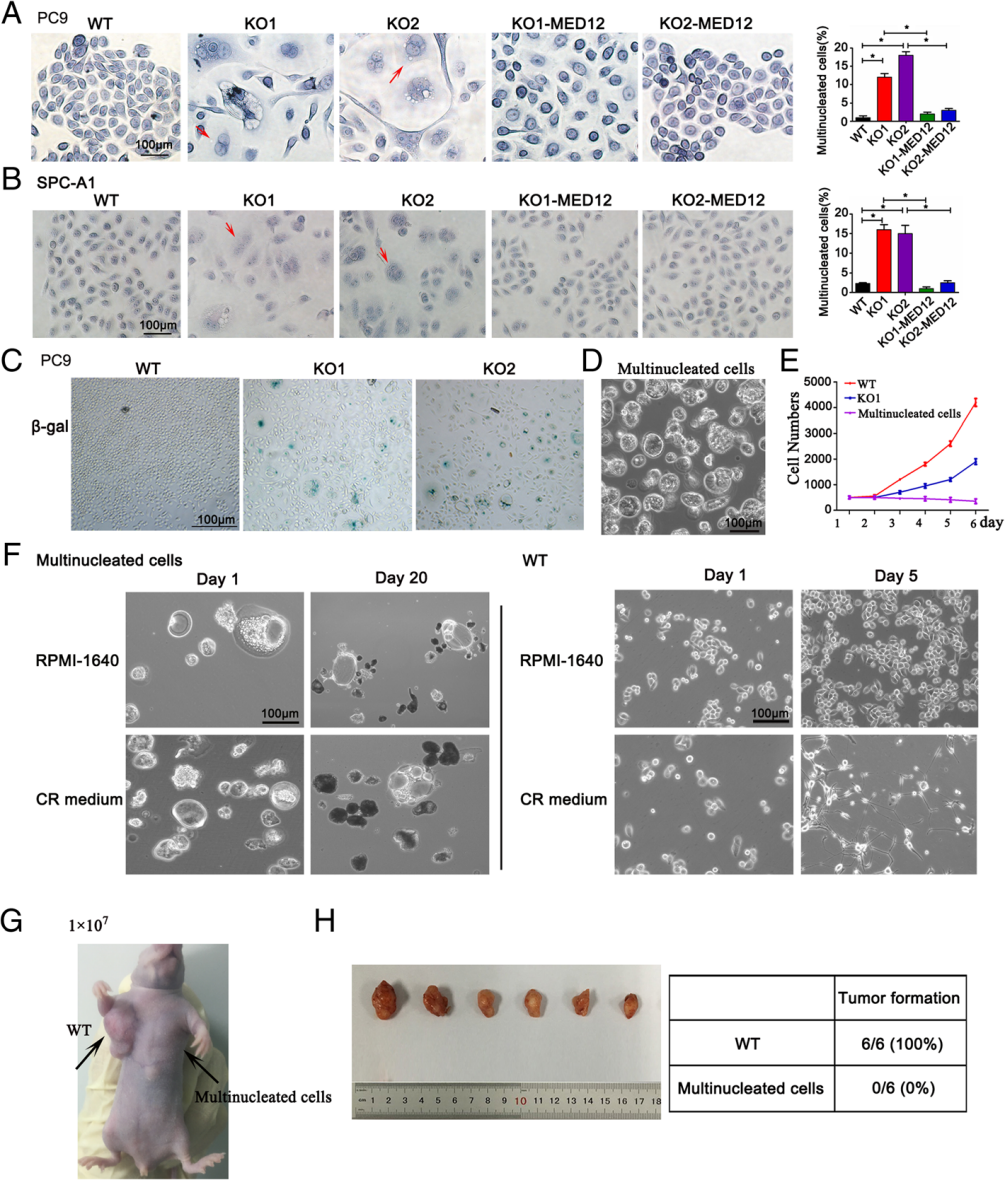
MED12 knockout caused a multinucleation phenotype in NSCLC cells. **a**-**b** HE staining showed the multinucleation in MED12 WT, MED12 KO and MED12 reconstitution single clones of PC9 and SPC-A1 cells. Arrowheads indicated multinucleated cells. Scale bar: 100 μm. *P < 0.05. **c** Expression of active β-galactosidase detected by X-gal staining. β-gal positive cells represent senescent cells. Scale bar: 100 μm. **d** Representative photograph of multinucleated cells sorted from MED12 KO1 of PC9 cells. Scale bar: 100 μm. **e** Cell counting assay in WT, KO1 and multinucleated cells sorted from MED12 KO1 of PC9 cells. **f** The survival of MED12 WT and multinucleated cells sorted from MED12 KO1 of PC9 cells in RPMI-1640 and conditional reprogramming (CR) medium. Scale bar: 100 μm. **g** Schematic of xenograft experiment in vivo. **h** Representative photographs of xenograft tumors at the end of the experiment

We found that MED12 knockout caused multinucleation in PC9 cells and restoring MED12 expression in MED12 KO cells was sufficient to avert multinucleation (Fig. 3a). A similar phenomenon was observed in SPC-A1 cells (Fig. 3b). Next, we investigated the cell fate of multinucleated cells and found that they exhibitedsenescence (Fig. 3c). To determine the viability of multinucleated cells directly, we sorted these multinucleated cells from MED12 KO1 of PC9 cells via 40 μm cell strainer and found that multinucleated cells lost the proliferation ability totally in vitro (Fig. 3d and e). Compared with MED12 WT cells, multinucleated cells could not survive even though cultured with conditional reprogramming (CR) medium which allowed the epithelial cells propagated indefinitely in vitro (Fig. 3f). Similarly, multinucleated cells lost the tumorigenic ability in nude mice (Fig. 3g and h)*.* Collectively, these results suggested that MED12 knockout resulted in a multinucleation phenotype in NSCLC cells and these multinucleated cells in MED12 KO clones lost viability in vitro and in vivo.

### Loss of MED12 induced multinucleation through cytokinesis defects

**Fig. 4:**
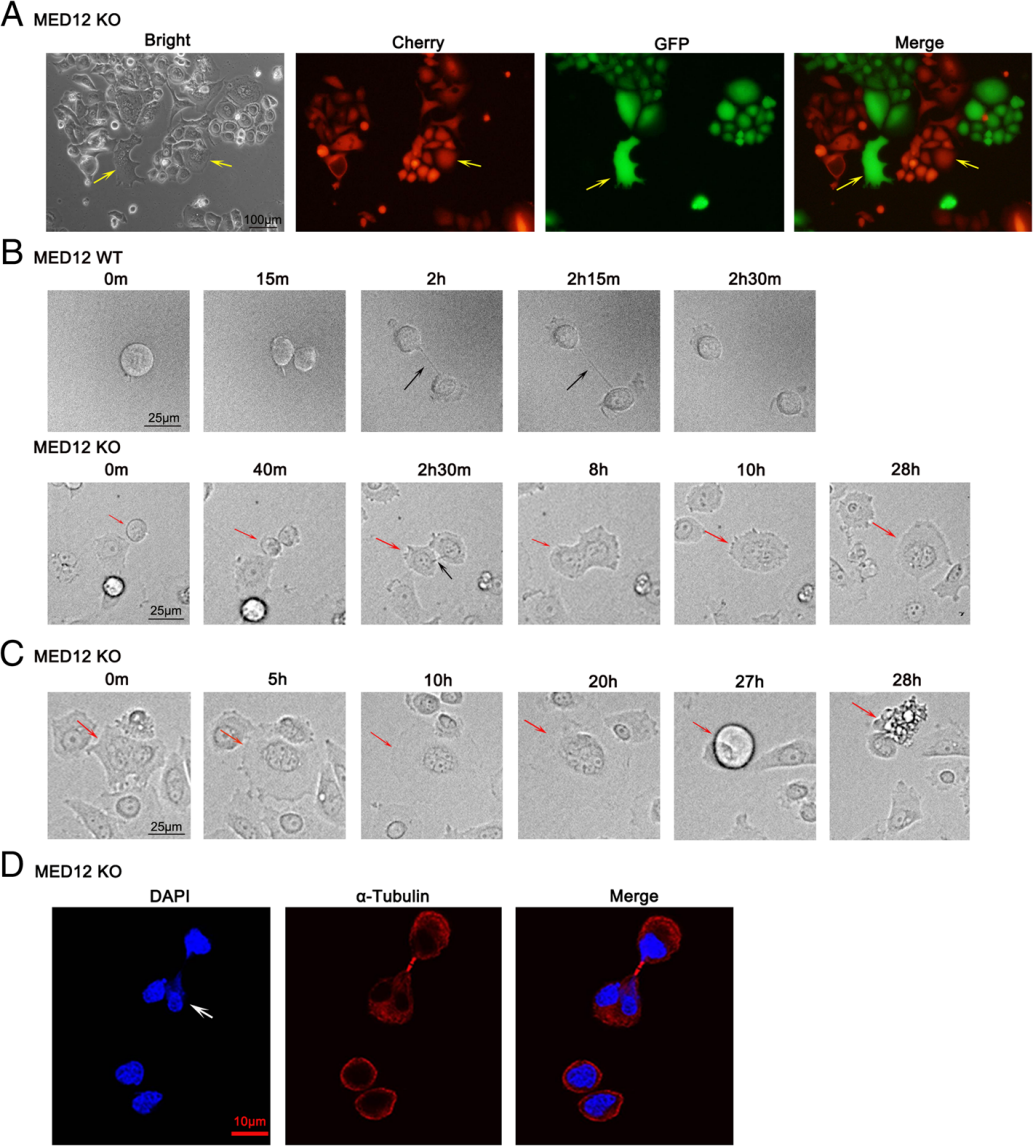
Loss of MED12 induced multinucleation through cytokinesis defects. **a** MED12 KO1 of PC9 cells stably expressing either Cherry-vector (red) or GFP-vector (green) were co-cultured. Representative micrographs were shown. Arrows indicated the multinucleate cells. Scale bar: 100 μm. **b** Live-cell images displayed the division process of WT and MED12 KO of PC9 cells. Black arrowheads indicated intercellular bridge. MED12 WT cell completed cytokinesis successfully in 2.5 h. The cytokinesis of KO1 cells were delayed and failed. Red arrowheads highlighted the cell which attempted to undergo cytokinesis. The two daughter cells remained arrested at the post midbody stage for about 7 h and failed to pass abscission and eventually rejoined one cell again with two nuclei. Scale bar: 25 μm. **c** Multinucleated cell didn’t pass to the next mitosis for 28 h and crashed eventually. Arrowheads indicated the multinucleated cell. **d** Immunofluorescence staining for nuclear atypia within MED12 KO cells during cytokinesis with alpha-tubulin and DAPI. Arrowheads denoted heteromorphic nucleus. Scale bar: 10 μm

Multinucleated cells could be the result of cell fusion event or failed cytokinesis. We therefore tested which event accounted for the formation of multinucleated cells following MED12 deletion. Firstly, MED12 KO1 of PC9 cells were transfected with Cherry-vector (red) or GFP-vector (green) prior to seeding co-cultures, respectively. After two weeks of co-cultures, the multinucleated cells appeared were only carrying either green or red, and no yellow (both) (Fig. 4a). This observation was incompatible with the former assumption that multinucleated cells were formed via cell-cell fusion. To confirm the latter hypothesis, we traced cell division process in plentiful fields by live cell imaging. Results showed that MED12 WT cells successfully completed cytokinesis in 2.5 h (Fig. 4b, Additional file 1: Movie S1). In contrast, MED12 KO cells were characterized by abscission failure of the intercellular bridge, and as a result, the dividing cell became multinucleated (Fig. 4b, Additional file 2: Movie S2). Many of the multinucleated cells did not pass to the next mitosis for a long time and crashed eventually (Fig. 4c, Additional file 2: Movie S2). Additionally, we observed the striking atypical nuclei formation within MED12 KO cells during cytokinesis (Fig. 4d). Taken together, multinucleated cells resulted from MED12 knockout was attributed to the failure of intercellular bridge abscission.

### MED12 knockout altered the expression of actin-relevant genes in NSCLC cells

**Fig. 5:**
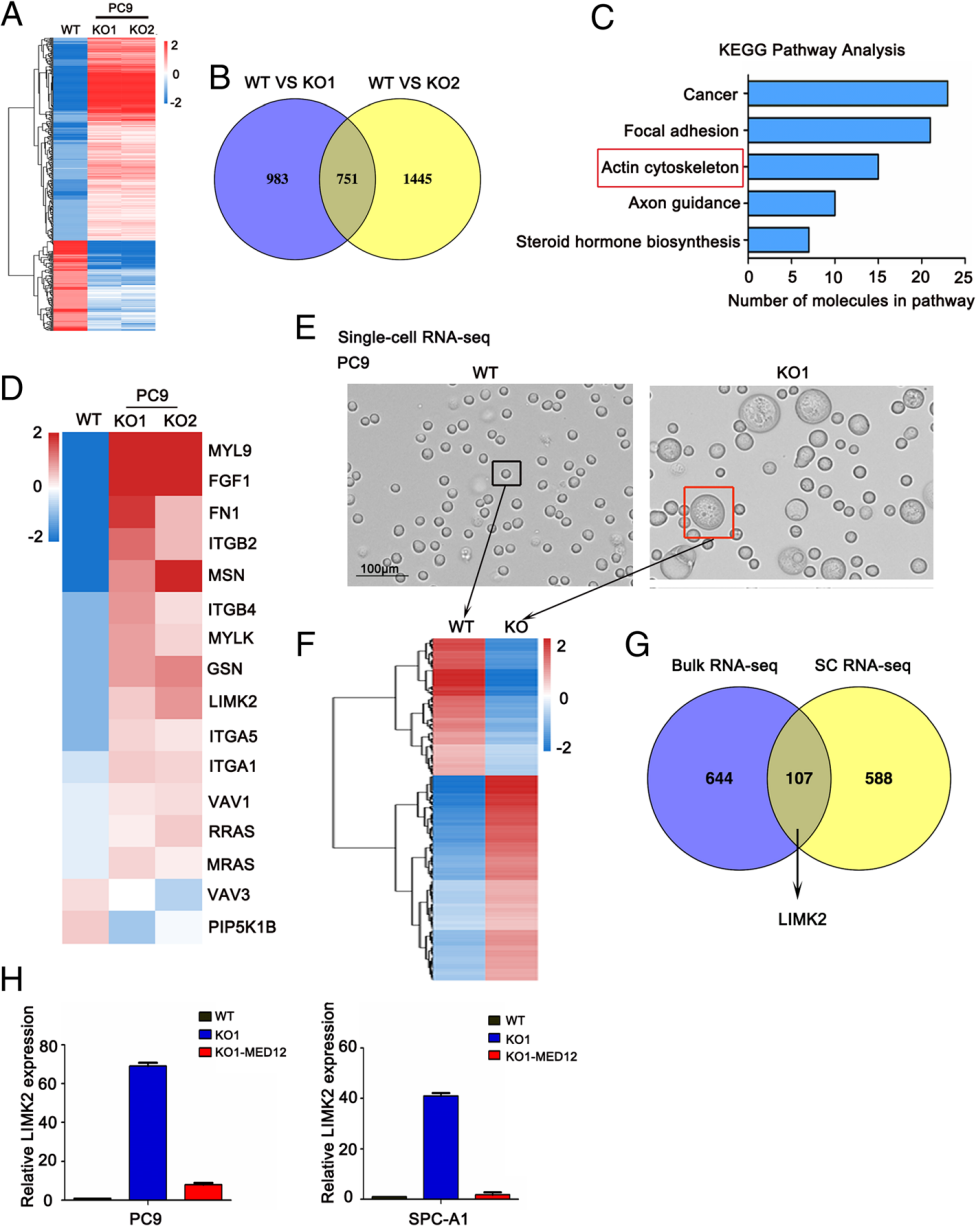
MED12 knockout altered the expression of actin-relevant genes in NSCLC cells. **a** Heat map of mRNA changes in WT and MED12 KO single clones of PC9 by bulk RNA-seq. **b** Venn diagrams depicted 751 common differential genes in MED12 KO single clones of PC9. **c** KEGG pathway assay of differential mRNA transcripts in MED12 KO clones identified by RNA-seq. **d** Heat map of mRNA changes in actin cytoskeleton by RNA-seq. **e** Representative images of single cell sorted from WT and MED12 KO1 of PC9 cells. Scale bar: 100 μm. **f** Heat map of mRNA changes in single cells sorted from WT and MED12 KO1 of PC9 cells by Single cell RNA-seq. **g** Venn diagrams depicted 107 common differential genes in bulk RNA-seq and Single cell RNA-seq. **h** Real-time PCR assay of LIMK2 in WT, MED12 KO1 and KO1-MED12 clones of PC9 and SPC-A1 cells

We applied bulk RNA-seq to further investigate the molecular mechanism of aborted cytokinesis resulted by MED12 knockout. Remarkably, bulk RNA-seq analysis identified 501 genes that were significantly up-regulated and 250 genes down-regulated in MED12 KO clones of PC9 cells by at least 2-fold with *p* < 0.05 (Fig. 5a and b). Pathway analysis identified the ‘actin cytoskeleton’, which is essential for cytokinesis, was dysregulated in MED12 KO cells (Fig. 5c). Total 16 genes including 14 up-regulated and 2 down-regulated genes involved in actin cytoskeleton pathway (Fig. 5d). To refine our understanding of which gene account for aborted cytokinesis in MED12 KO cells, we also performed single-cell RNA sequencing (scRNA-seq) in giant multinucleated cells isolated from MED12 KO clones of PC9 cells (Fig. 5e and f). Consistently with our bulk RNA-seq results, 107 genes including actin-relevant gene LIMK2 was screened out (Fig. 5g). Real-time PCR assay verified that LIMK2 was up-regulated in MED12 KO cells and restored after reconstitution of MED12 (Fig. 5h). These results indicated that cytokinesis defect caused by MED12 knockout was probably due to disorganization of actin cytoskeleton, and which perhaps was related to LIMK2 mainly.

### MED12 regulated the balance of F-actin polymerization and depolymerization via LIMK2/cofilin pathway in NSCLC cells

**Fig. 6:**
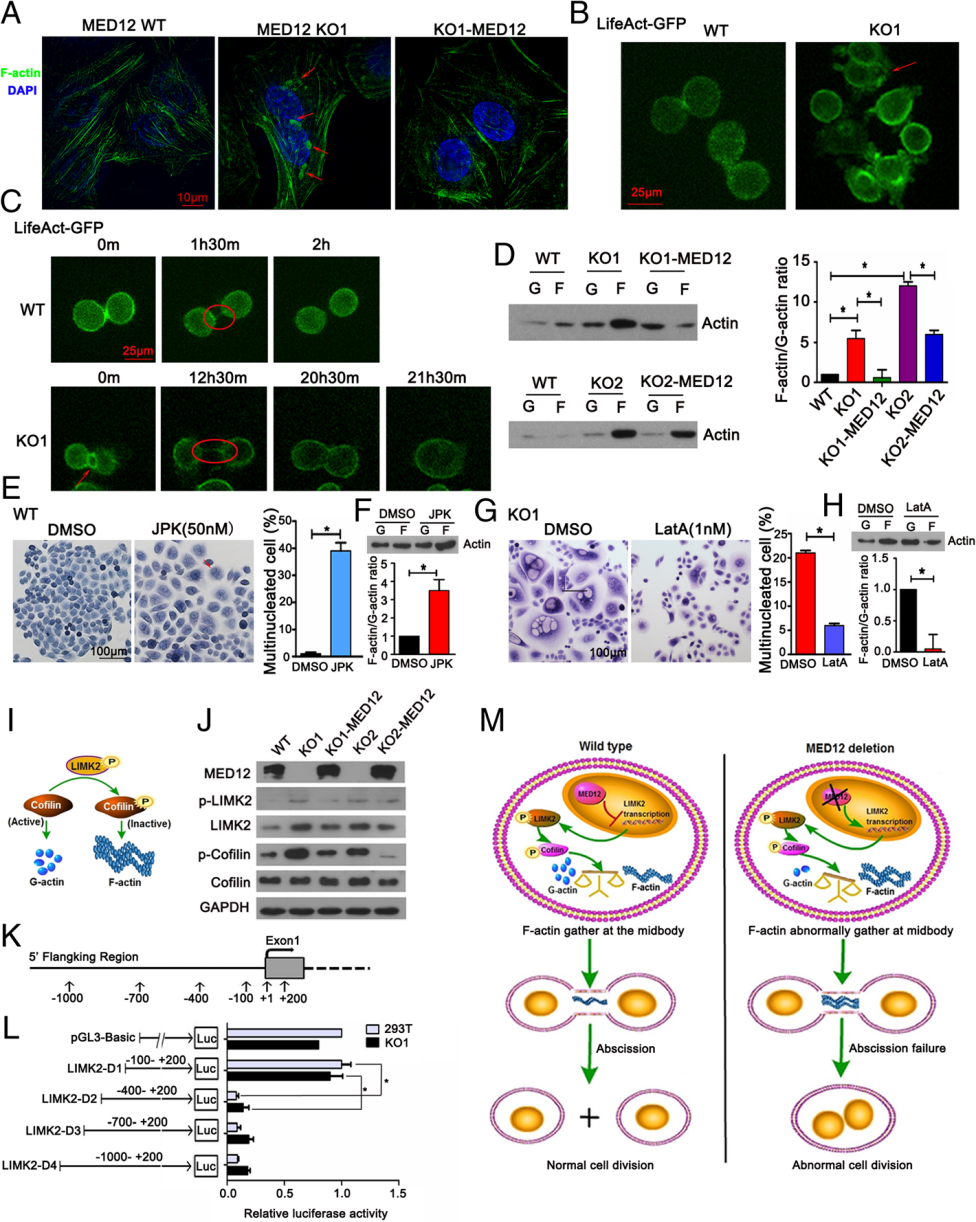
MED12 regulated the balance of F-actin polymerization and depolymerization via LIMK2/cofilin pathway in NSCLC cells. **a** F-actin was detected using FITC-phalloidin. Representative micrographs were shown. Nucleus was counterstained by DAPI. Arrowheads indicated F-actin location abnormally. Scale bar: 10 μm. **b** Live-cell images displayed the arrangement of F-actin cytoskeleton. Red rectangle indicated disordered F-actin cytoskeleton of MED12 KO1 cells stably expressed pLifeAct-TagGFP2. Scale bar: 25 μm. **c** Live-cell images displayed the cytokinesis process of MED12 WT and MED12 KO1 of PC9 cells stably expressed pLifeAct-TagGFP2. MED12 KO1 cell was unable to pass abscission of intercellular bridge and eventually rejoined to one cell again. Arrowheads indicated F-actin abnormally located at the contractile ring and ellipses indicated the intracellular bridge. Scale bar: 25 μm. **d** Western blotting assay of G-actin and F-actin expression and quantification of the F-actin/G-actin ratio in WT, MED12 KO and MED12 reconstitution single clones of PC9 cells. *P < 0.05. **e** HE staining showed multinucleation in control and JPK-treated MED12 WT of PC9 cells. Arrowhead indicated multinucleated cell. Scale bar: 100 μm. *P < 0.05. **f** Western blotting analysis of G-actin and F-actin expression and quantification of the F-actin/G-actin ratio in control and JPK-treated MED12 WT of PC9 cells. *P < 0.05. **g** HE staining showed multinucleation in control and LatA-treated MED12 KO1 cells. Arrowhead indicated multinucleated cell. Scale bar: 100 μm. **h** Western blotting analysis of G-actin and F-actin expression and quantification of the F-actin/G-actin ratio in control and LatA-treated MED12 KO1 of PC9 cells. *P < 0.05. **i** Schematic of the function of LIMK2/cofilin in actin assembly. **j** Western blotting assay of LIMK2/cofilin pathway in WT, MED12 KO and MED12 reconstitution single clones of PC9 cells. **k** Map of the 1200 bp test promoter region relative to *LIMK2* gene. **l** Dual-luciferase reporter analysis verified the targeting relationship between MED12 and *LIMK2* in HEK-293FT and MED12 KO1 of PC9 cells. A series of deleted constructs containing the individual sequence D1- D4 were designed. Values are expressed as means ± SE of three replicates. *P < 0.05. **m** The schematic describes a proposed model for the role of MED12 in the regulation of actin dynamics and intracellular bridge abscission during cytokinesis, and that MED12 deletion resulted in aborted cytokinesis

To test the hypothesis above, we first labeled the F-actin filament of WT, KO1 and KO1-MED12 of PC9 cells with FITC-phalloidin. Compared with WT and KO1-MED12 cells, MED12 KO1 cells exhibited with F-actin aberrant localization (Fig. 6a). Furthermore, we applied time-lapse microscopy to observe spatiotemporal changes of the actin cytoskeleton in cells transfected p^CMV^-LifeAct-TagGFP2, which was used to identify F-actin [22]. We found that the arrangement of F-actin cytoskeleton occurred orderly in MED12 WT cells, but was clearly disorganized in MED12 KO cells (Fig. 6b, Additional file 3: Movie S3 and Additional file 4: Movie S4). Live cell imaging showed that F-actin was chaotic positioned on equatorial furrowing and was unsuccessfully removed from the intercellular bridge in MED12 KO1 cells (Fig. 6c, Additional file 3: Movie S3 and Additional file 4: Movie S4). Next, we examined the ratio of F-actin to G-actin in WT, MED12 KO and MED12 reconstitution single clones of PC9 cells which was on behalf of the balance between F-actin polymerization and depolymerization. We observed a potent increase in the ratio of F-actin to G-actin in MED12 KO cells, and re-expression of exogenous MED12 prevented the accumulation of F-actin (Fig. 6d). We next investigated whether the balance between actin polymerization and depolymerization was essential for normal execution of cytokinesis. Jasplakinolide (JPK) has proven to be able to promote F-actin polymerization [23]. After JPK stimulation, multinucleated cells were observed in MED12 WT of PC9 cells and the F-actin polymerization was increased (Fig. 6e and f). To directly test whether multinucleation induced by MED12 knockout was due to F-actin abnormal polymerization, MED12 KO1 of PC9 cells were treated with LatrunculinA (LatA) which could reduce F-actin polymerization. The result showed that LatA reduced F-actin polymerization and slightly reduced the ratio of multinucleated cells (Fig. 6g and h). These results implied that MED12 acted as a negative regulator of F-actin polymerization for accurate cytokinesis.

RNA-seq assay revealed that the LIMK2 mRNA was upregulated in MED12 KO cells (Fig. 5g). It was reported that the function of LIMK2 in actin assembly was related to cofilin phosphorylation levels (Fig. 6i). Our results demonstrated that the upregulated LIMK2 led to the acceleration of its phosphorylation in MED12 KO cells, and activated LIMK2 significantly increased the phosphorylation of its downstream molecule cofilin (Fig. 6i). Furthermore, we found that phosphorylation of LIMK2 and cofilin significantly decreased after reconstitution of exogenous MED12 in MED12 KO cells (Fig. 6i). To determine whether the effect of MED12 on LIMK2 expression was due to direct transcriptional inhibition of the *LIMK2* gene, we performed dual-luciferase reporter assay (Fig. 6j). The result showed that MED12 expression repressed the transcriptional activity of *LIMK2* by occupying on the *LIMK2* promoter and − 400 bp to − 100 bp was essential for its transcriptional activity (Fig. 6k and l). Collectively, these results indicated that MED12 knockout disturbed the balance between F-actin polymerization and depolymerization via activating LIMK2/cofilin signaling.

## Discussion

The results from the current study unveiled a crucial role of MED12 in cytokinesis of NSCLC cells (Fig. 6m). Our data demonstrated that higher MED12 expression correlated with larger tumor volume in NSCLC patients and aborted cytokinesis in MED12 KO cells induced cells senescence and suppressed cell proliferation both in cell culture and mouse xenografts. Also, we showed that MED12 promoted cytokinesis through modulating the balance of F-actin polymerization and depolymerization, ensuring an effective completion of cellular division in highly proliferative NSCLC cells. This observation that cell division of NSCLC depends on MED12 promises future exploitation by targeting MED12 as a cytostatic cancer therapy.

Cytokinesis failure can be divided into the two following cases: early cleavage apparatus defects and terminal abscission defects [24–26]. As shown in this study, MED12 knockout did not cause aberration in early positioning of the cleavage furrow and the actomyosin ring contract but resulted in incomplete partitioning of daughter cells by impeding the final abscission. This implies that MED12 act as a regulator of late cytokinesis to make sure cells progressed through division normally. Incomplete cytokinesis produced by MED12 knockout contributed to the formation of multinucleated cells which had the feature of senescence and lost viability. Previous studies demonstrated that cells treated with drugs blocking cytokinesis would undergo senescence and cell death [27–29]. These studies further support our conclusion that MED12 plays an important role in regulating cell cytokinesis in NSCLC cells. Whether MED12 has the similar function in other cancer cells is currently under investigation by our laboratory.

Reorganization of cell cytoskeleton plays a vital role in cytokinesis and actin stress fibers are one of the major cytoskeleton structures [30–32]. MED12 knockout led to abnormal activation of the actin-relevant genes which further disturbance the remodeling of actin cytoskeleton. The flattening and scattering phenotype of MED12 knockout cells was in accordance with the cell morphology changes caused by the disorganization of the actin cytoskeleton. Previous study has shown that removal of F-actin accumulation in remnants of the midbody was a prerequisite for abscission [33]. Apropos to this observation, our live cell imaging well showed that F-actin abnormally gathered and was unsuccessfully removed from the intercellular bridge in MED12 KO cells. These results indicated that chaotic position of F-actin could account for the defective abscission observed in NSCLC cells after MED12 knockout.

The intercellular bridge offers a binding platform for abscission factors which is essential for normal abscission [34]. Proteomic analysis of isolated intercellular bridges authenticated a great variety of protein components are necessary for completion of cytokinesis, a large number of which belong to actin associated genes [35]. In addition, recent studies have indicated that abscission components could be able to recruit, and the intercellular bridge would then split only when membrane vesicle transported and inserted for the closure of the intercellular channel accurately [36–38]. Precise temporal and spatial regulation in assembly and disassembly of actin filament is essential for intracellular vesicle transport and membrane addition [39]. Our study showed that multinucleation was accompanied by large abnormal vacuoles in MED12 KO cells, which might be resulted from perturbation of vesicle trafficking or membrane vesicle fusion with the cells. Even though we have known that failure of cytokinesis induced by MED12 knockout was due to the disability of abscission caused by chaotic position of F-actin, it is not clear whether this fault is also attributed to disrupting recruitment of abscission factors or disrupting vesicle trafficking and insertion for closure the space remaining intercellular bridge. In future work, it will be important to reveal these mysteries.

MED12 acts as a transcriptional hub, regulates gene expression by interacting with specific transcription factors [40]. Developmental transcription factors including β-catenin interact with MED12 to help determine cell fate [41, 42]. Our RNA-seq assay showed that most of differential genes were upregulated in MED12 KO cells. This finding was consistent with a previous study that MED12 was originally thought to act as a transcriptional repressor [43]. LIMK2, a member of the LIMK serine/threonine protein kinase family, mediates multiple cellular processes including cytokinesis. It was reported that the kinase LIMK2 could modulate the dynamics of actin polymerization via phosphorylates and inactivates the actin-depolymerizing factor cofilin [16, 17]. In this study, Real-time PCR assay showed that the mRNA expression of LIMK2 was upregulated in MED12 KO cells and potently restored after reconstitution of MED12, suggesting a transcriptional regulatory mechanism was present between MED12 and LIMK2. And then, we verified that MED12 could bind to the promotor of LIMK2 and inhibited the transcription of LIMK2. Increased and activated LIMK2 also phosphorylated cofilin in our study. These results suggested that MED12 exerts transcriptional regulation role in LIMK2/cofilin pathway.

## Conclusions

In summary, our findings provide the first cogent phenotypic and molecular evidences that MED12 was indispensable for actin-mediated cytokinesis partly via LIMK2/Cofilin pathway in NSCLC cells. Cells entrance into senescence and loss viability due to aborted cytokinesis represents an important strategy to control tumor growth. Therefore, this study offers a rationale that MED12 mediated cytokinesis could be a target for NSCLC especially those highly expressed MED12.

## Materials and methods

### Reagents and chemicals

General laboratory reagents were obtained from Thermo Fisher Scientific and Sigma-Aldrich. Commercially available antibodies against MED12 (ab70842) and actin (ab205) were obtained from Abcam (USA). The antibodies against α-Tubulin (T9026), LIMK2 (SAB4501760), p-LIMK2 (SAB4300104), cofilin (SAB2702206,) and p-cofilin (SAB4504370) were obtained from Sigma-Aldrich. Jasplakinolide (JPK) and latrunculinA (LatA) were purchased from Sigma-Aldrich.

### Patient information and tissue specimens

Paraffin-embedded tissues were obtained from 179 patients with NSCLC who were treated at Sun Yat-sen University Cancer Center between January 2010 and January 2011. The chosen criteria used for patient enrollment in the cohorts based on the following: do not receive chemotherapy or radiotherapy prior to surgical resection, do not combine other tumors during the five yeas follow-up period, died from tumor and the availability of follow-up data. The study was ratified by the Review Board of Sun Yat-sen University Cancer Center. Written informed consents were obtained from all enrolled patients.

### MED12 mutation screening

Genomic DNA from each sample was extracted using DNeasy blood and tissue kit (Qiagen). The MED12 DNA fragment was amplified by polymerase chain reaction (PCR) with three primer pairs (5′- GTGAAATCGACTTGCTGCCG -3′ and 5′- GCAAAGCTCGTGATCTGCTC -3′, product size: 8000 bp; 5′- TCTCACTCCCACTGCCCTTA -3′ and 5′- CGGGTGGGGATGCTTTACTT -3′, product size: 8200 bp; 5′- GGAGATCATCATCAGCGGCA -3′ and 5′- AGACATGCAGAACTCGCACA -3′, product size: 8100 bp). After amplification, PCR products were purified using QIAquick PCR Purification Kit (Qiagen) and evaluated by ethidium bromide staining on a 1% agarose gel. The sequencing reactions were performed utilizing the NEBNext Ultra DNA Library Prep Kit for Illumina (NEB #E7370S/L) according to the manufacturer’s instructions. The reactions were then run on Illumina MiSeq sequencing platform. The mutation was analyzed using bwa software. NM_005120.2 from NCBI was used as reference sequence.

### Immunohistochemistry (IHC)

Paraffin-embedded tissues were sectioned, mounted on positively charged glass slides, baked, deparaffinized, and rehydrated. Antigen retrieval was completed by heating slides in EDTA (pH 8.0) for 10 min in a pressure cooker. Sections were incubated with MED12 antibody overnight at 4 °C, and then incubated with the secondary antibodies at 37 °C for 45 min (Dako Envision). Sections were stained with diaminobenzidine (DAB) and visualized with an Eclipse 80i (Nikon, Japan). Immunostaining assay was performed by two observers independently according to both the intensity of staining and the proportion of staining. The H-score was calculated as the score of staining intensity (negative, 0; weak, 1; moderate, 2; or strong, 3 scores) × score of positive tumor cells proportion (≤25%, 1; 26–50%, 2; 51–75%, 3; > 75%, 4 scores). MED12 expression in specimens was divided into low expression (H-score ≤ 8) and high expression (H-score > 8) groups according to a cutoff value of the ROC curve.

### Cell lines and cell culture

The human NSCLC cell lines (PC9, SPC-A1, A549, H1299, H1975 and GLC-82) and human embryonic kidney cells (HEK-293FT) were all purchased from the ATCC. PC9, A549, H1299 and H1975 cells were grown in RPMI 1640 medium. SPC-A1, GLC-82 and HEK-293FT cells were grown in Dulbecco’s modified Eagle’s medium (DMEM) medium supplemented with 10% fetal bovine serum and 100 IU/ml penicillin/streptomycin (Sigma). All cells were grown at 37 °C in a humidified 5% CO_2_ atmosphere.

### Generation of MED12 knockout cell lines

MED12 knockout (KO) cell lines were generated using the CRISPR–Cas9 system. The Cas9 plasmids (#53373) and pGL3-U6-sgRNA vector (#51133) were purchased from Addgene. The sgRNA1 sequence ACGGCCTTGAATGTAAAACA targeting exon2 was used to generate the MED12 knockout clone MED12 KO1 and the sgRNA2 sequence GGCTAGTTGCGTGAGTGGCT targeting exon3 was used to generate the MED12 knockout clone MED12 KO2. PC9 or SPC-A1 cells were transfected with 1 μg *MED12* sgRNA plasmid plus 1 μg Cas9 plasmid. Following 5 μg puromycin selection for two days, cells were sorted into 96-well plates. Single-cell clones were expanded and validated as MED12 knockout clone by Western blotting and Sanger sequencing.

### Rescue expression of MED12

MED12 CDS fragment which amplified from a pAc-C Med12His plasmid (Addgene plasmid #49240) was cloned into the pLenti6/V5-D-TOPO vector (Invitrogen) at the BamH1 and Xho1 sites. Lentivirus was produced by transfection of HEK-293FT cells with pLenti6-MED12, psPAX2 and pMD2.G plasmid at a 0.5:0.35:0.15 ratio. The viral supernatant was collected 48 h following transfection, filtered through 0.45 μm filter, and added to MED12 KO1 and MED12 KO2 cells. The stably expressing cell lines were selected with 20 μg/ml blasticidin for 5 days and named as KO1-MED12 or KO2-MED12.

### Stable transfection

To stably knock down endogenous MED12 expression, cells including A549, H1299, H1975 and GLC-82 were grown to 30% confluency in 6-well culture plates and then infected with lentivirus carrying an shRNA targeting MED12 (ShMED12) or a negative control vector (ShCtrl) (Sigma). After 48 h of infection at 37 °C, the medium was replaced with fresh medium and incubated further for 72 h before analysis using RT-qPCR and western blotting for MED12 expression. The shRNA sequences used were 5′-GGTACTTCATACTTTGGAA -3′ (ShMED12^1#^) and 5′-AGAGAAATTACGTTGTAAT -3′ (ShMED12^2#^).

### Western blotting

Cells were lysed with cold RIPA supplementing with protease inhibitors (Beyotime Biotechnology). Pierce BCA assay determined the protein concentrations (Thermo Fisher Scientific). The interest proteins were separated by SDS–PAGE and transferred onto PVDF membranes (Millipore, USA). These membranes were then blocked with 5% nonfat milk, incubated with primary antibodies overnight at 4 °C and secondary antibodies for 2 h at room temperature. Protein–antibody complexes were detected by ECL (Bio-Rad).

### H&E staining

The hematoxylin and eosin (H&E) (Beyotime Biotechnology) was applied to stain for nuclei and cytoplasm respectively according to manufacturer’s instructions.

### Cell proliferation assay

The methyl thiazolyl tetrazolium (MTT) colorimetric assay was used to detect cell viability. Briefly, cells were planked in 96-well plates with a density of 1000 cells per well. At indicated time points, the cell supernatant was added 20 μl of MTT (5 mg/ml) and then incubated for a further 4 h. Thereafter, the medium was removed, 150ul dimethyl sulfoxide (DMSO) was added into each well to dissolve the formazan precipitate. The absorbance at a wave length of 490 nm was measured by a microplate reader (BIO-RAD, Hercules, CA, USA).

Colony formation assay, cells were seeded in six-well plates (500 cells/well). After incubation for 10 days, the colonies were fixed with 4% paraformaldehyde, and stained with 0.2% (w/v) crystal violet. Colonies were quantified with the Image J software.

### Xenograft tumor assay

All in vivo experiments were performed following guidelines for the use of laboratory animals of the Sun Yat-sen University Institutional Animal Care and Use Committee. Nude mice (NU/NU, 4–5 weeks of age, female, 15-17 g) were purchased from the Beijing Vital River Laboratory Animal Technology Co., Ltd. (Charles River Laboratories). The experimental cells were resuspended in 200 μl PBS and injected subcutaneously into the left or right dorsal flank of 8 female nude mice. The two perpendicular maximum diameter (A) and minimum diameter (B) of tumor were measured every 4 days, and tumor volume (V) was calculated as formula: V = 0.5 × A × B × B. Experimental mice were euthanized at the end of the observation period, and then the tumors were excised and weighed.

### Live cell imaging

Cells in a 35 mm glass-bottom Petri dish (Corning) were placed in microscope chamber with a 37 °C atmosphere of 5% CO_2_ and observed under differential interference contrast. Images were acquired every 5 min with YOKOGAWA CV1000, equipped with × 40 dry lens using the auto time series Macro with autofocus. GFP fluorescence was excited with the 488-nm line of an argon ion laser.

### Immunofluorescence

Cells grown on cover slips, were fixed with 4% paraformaldehyde, permeabilized in 0.1% Triton-X-100, and blocked with 5% BSA. For F-actin localization, the cells were stained with FITC-phalloidin (1:1000) solutions for 20 min at room temperature. For tubulin staining, cells were incubated with the α–tubulin primary antibody (1:300) for 2 h, and then stained with Alexa Fluor®633-conjugated secondary antibody for another 2 h. Cell nucleus was showed by DAPI. Images were captured with a FV1000-D confocal laser scanning microscope carrying a UPLSAPO 100× NA 1.4 oil immersion lens (Olympus).

### Senescence-associated β-galactosidase assay

SA-β gal staining was performed in cultured cells with the senescence staining kit following the manufacturer’s recommendation (Beyotime Biotechnology). Images were taken with an inverted microscope (Olympus).

### F-actin/G-actin ratio

Briefly, the difference between two forms of actin is that G-actin is soluble, while F-actin is insoluble. The cells were first lysed in cold lysis buffer1 (50 mM KCl, 100 mM NaF, 10 mM K2HPO4, 2 mM MgCl2, 0.2 mM DTT, 1 mM EGTA, 1 mM sucrose, 0.5% Triton X-100, pH 7.0), and then centrifuged for 30 min at 14,000×g. G-actin was soluble in the supernatant. The pellet contained insoluble F-actin was resuspended with an equal volume of lysis buffer 2 (20 mM Tris-HCl, 1 mM sodium acetate, 1.5 mM guanidine hydrochloride, 1 mM CaCl2, 1 mM ATP, pH 7.5,) which converted F-actin into soluble G-actin. The samples were incubated on ice for 1 h with gentle mixing every 10 min and then centrifuged at 14,000×g for 30 min so as to F-actin could be measured in this supernatant. Equal volumes of F-actin and G-actin were loaded and then analyzed using Western blotting by anti-actin antibody. Densitometry analysis was done using Image J.

### Bulk and single-cell RNA transcriptomes sequencing (RNA-seq) assay

Total RNAs from experimental cells were extracted by an RNeasy plus mini kit (Qiagen) according to the manufacturer instruction. A total of 2 ng purified cDNA products from each sample were used for library construction. All of the samples were sequenced on Illumina HiSeq 2000 sequencing system.

### Luciferase reporter assay

Four *LIMK2* promoter deletion fragments (D1: − 100 bp to + 200 bp, D2: − 400 bp to + 200 bp, D3: − 700 bp to + 200 bp and D4: − 1000 bp to+ 200 bp) were cloned into pGL3-Basic vector (Promega) to determine the core promoter region. The pGL3-LIMK2 promoter luciferase construct and pRL-TK (Promega) coding for Renilla were co-transfected with MED12 expression vector or negative control into HEK-293FT or MED12 knockout of PC9 cells using Lipofectamine 2000. Luciferase activity was determined 48 h after co-transfection using the Dual-Luciferase Reporter Assay System (Promega). Luciferase activities were normalized to the expression of Renilla and presented in ratios of Firefly to Renilla luciferase reporter activities.

### Statistical analysis

Results were depicted as mean ± SD, each in vitro experiment was repeated at least three times. Statistical analysis was done with Prism GraphPad software v5.0 (San Diego, CA, USA). Two-tailed Student’s t-test was used for statistical comparison between groups and a difference was considered significant if *p* < 0.05. The chi-squared test was applied to examine the correlation between MED12 expression and clinical pathological parameters. *P < 0.05.

## Additional files

Additional file 1: **Movie S1.** Loss of MED12 induced cytokinesis defects. Live cell imaging showed that MED12-WT cells successfully completed cytokinesis (Related to Fig. 4b). (AVI 23958 kb)

Additional file 2: **Movie S2.** Loss of MED12 induced cytokinesis defects. The time-lapse images of cytokinesis defects in MED12-KO1 cells (Related to Fig. 4b-c). (AVI 260956 kb)

Additional file 3: **Movie S3.** MED12 knockout altered the rearrangement of actin cytoskeleton. The rearrangement of actin cytoskeleton occurred normally in MED12-WT cells transfected with LifeAct-GFP (Related to Fig. 6b-c). (AVI 385016 kb)

Additional file 4: **Movie S4.** MED12 knockout altered the rearrangement of actin cytoskeleton. MED12-KO1 LifeAct-GFP cells exhibited with destroyed cell shape and disorganized actin cytoskeleton (Related to Fig. 6b-c). (AVI 256678 kb)

## Abbreviations

CR: Conditional reprogramming
CRISPR: Clustered Regularly Interspaced Short Palindromic Repeats
IHC: Immunohistochemistry
KO: Knockout
MED12: Mediator complex subunit 12
Med8: Mediator complex subunit 8
NSCLC: Non-small cell lung cancer
OS: Overall survival
PFS: Progression-free survival
Pol II: Polymerase II
